# Perceived Family Function and Associated Predictors in Nurses: A Cross-Sectional Study

**DOI:** 10.3389/fpsyt.2022.904581

**Published:** 2022-06-20

**Authors:** Wen Zeng, Qian Fang, Cui Wang, Beibei Tong, Dan Li, Ziqiu Zou, Peiyuan Liu, Yuanrong Yao, Shaomei Shang

**Affiliations:** ^1^School of Nursing, Health Science Center, Peking University, Beijing, China; ^2^Guizhou Provincial People's Hospital, Guiyang, China

**Keywords:** determinant, family function, nurse, predictors, cross-sectional study

## Abstract

**Background:**

Nurses play a key role in the health care system. However, clinical nurses experience different kinds of stressors that might impact the nurses' quality of life or quality of care. Family is one of the main social support resources, and quality family function might improve the quality of care provided by nurses. However, evidence on family function in Chinese clinical nurses is quite limited.

**Objectives:**

The current study was to evaluate the family function of the Chinese clinical nurses, and to explore associated predicting factors.

**Methods:**

A multi-center cross-sectional anonymous online survey was carried out. Chinese Family Function Scale was used in the study. Spearman's rank correlation analysis, Mann-Whitney U test, or Kruskal-Wallis H test was performed in the univariate analysis. The pairwise comparison method was used to determine whether the difference was significant between pair groups. Categorical regression (optimal scaling regression) was the main method to analyze factors that had been confirmed to be statistically significant in the univariate analysis.

**Results:**

Nineteen thousand four hundred and twenty-two nurses completed the online questionnaires. The median of the nurse's perceived family function score was three (Inter-quartile Range: IQR 2–5). The multivariate analysis showed that the highest education level (*P* <0.001), the hospital level (*P* <0 .001), rotation shift status (*P* <0.001), working department (*P* < 0.001), number of children (*P* < 0.001), monthly income per family member (*P* < 0.001) were significantly associated with family function. Moreover, the importance of the factors was the number of children (49.1%), monthly income per family member (20.7%), rotation shift status (12.4%), the highest education level (8.0%), the hospital level (7.6%), and working department (2.4%) in turn.

**Conclusions:**

The family function was associated with multiple factors, which hints that managers, leaders, and government could make strategies to improve nurses' family function in order to lead nurses to make a balance between family and work. Policymakers, nursing managers, and employers should make strategies such as promoting children-care services, increasing nurses' income, educating and training enough nurses, and building a well-established system of career development to help clinical nurses improve their family function so that to improve the quality of care.

## Introduction

Family, as the basic social characteristic, provides material and spiritual support for family members. Therefore, family plays a core role in individual physical and mental health. Family function (FF), which was proposed by researchers in the 1970s (1), was originally studied in children. As the research goes on, different theories about FF have been developed. The definition differs depending on various theories. According to previous studies, FF was defined as “the ability of families to coordinate and adapt the changes throughout life, resolve the conflict, cooperate between members and success in disciplinary patterns, respect the boundaries between individuals and respect the rules and principles which help the family to protect the entire family system” (2). From this perspective, FF is a concept with multiple dimensions encompassing family roles, duties as well as functions (3). Particularly, FF involves the whole process of communicating, solving problems, labor dividing, managing conflict, and attaching emotions between family members (4).

In recent years, FF has received increased attention in both psychological and medical research areas. A growing number of studies have focused on FF in different subjects, for instance, male cancer patients (5), groups with or without diabetic women (3), patients with traumatic brain injury (6), children with asthma (7), adolescents (8), children with chronic conditions (9), first-episode psychosis (10), pediatric obsessive-compulsive and related disorders (11), school-aged children with Sickle Cell Disease (12), adolescents left behind by migrating parents (13), patients with stroke (13–15), elderly patients with hypertension (16), or patients with blood diseases (17). Based on demonstrable evidence of FF, quite a lot of those studies indicate that FF was largely associated with individuals' health conditions. For example, a meta-analysis carried out by Leeman et al. (9) demonstrated that good FF was deemed relevant with positive outcomes such as better quality of life, competence, adherence, and physical health. Another study revealed that adolescents with a higher level of FF got higher scores in perceived subjective well-being (8). In the study of Bennich et al. (18), there was a correlation between healthy FF and less burden of diabetes and better quality of life. Conversely, family dysfunction or adverse FF was linked with some psychological disorders or unfavorable health outcomes. For instance, Murphy and Flessner (11) conducted a literature review on FF in pediatric obsessive-compulsive and related disorders and found that poor FF might be a potential or maintaining factor in such a group. Some scholars applied structural equation modeling to demonstrate that mental disability was directly affected by FF in patients with schizophrenia (19).

In the healthcare system, nurses play a core role and encompass the main workforce in any healthcare organization. According to the statistics of the World Health Organization (WHO), there are 43.5 million health workers worldwide, while nurses are more than 20 million (20). Notably, more than 4 million registered nurses are in China (21). In other words, Chinese nurses count for nearly one-fifth of global nurses. Nurses deliver diverse healthcare services in different settings as healthcare providers, protectors, communicators, coordinators, decision-makers, and teachers (22). In fact, nurses are usually the first responders to different health-related conditions to promote health and rehabilitation and prevent disease (22).

Nurses, as a special professional group, play a vital role in the health care system. However, the job of a nurse is perceived as one challenging and stressful profession with high and complicated physical and psychological demands (23). In fact, nurses experience a high level of stress due to various dimensions of stressors such as death and dying stressors, discrimination, excessive workload especially frequent rotation shift working, conflict with a physician, patient, and workmates, which might significantly impact the quality of life of nurses, even worse, that might lead to negative quality of care (23). Family is one of the most important social support resources (24) for nurses to cope with such stressors. Therefore, the level of FF is of great concern. Appropriate FF plays a vital role in ensuring and promoting nurses to deal with occupational stress, which may promote job efficiency and quality of care (25). On the contrary, dysfunction of the family may increase the stress for nurses so that nurses could hardly deal with conflict from family and work (25, 26).

Despite of advances in studies on FF, to the best of our knowledge, research on FF as well as its predictors in nurses has been quite limited to date. Only a few studies evaluating nurses' FF were based on surveys with small sample sizes or in specific settings. In the study of Sun et al. (26), researchers investigated 124 ICU nurses working at a military hospital in Beijing, China, and they found that 56.45% of the investigated nurses were in families with good function. However, nearly 40% of nurses lived in a family with moderate dysfunction, even 4% with severe dysfunction. Similarly, Deng et al. (27) assessed 184 registered nurses who worked at a surgical department. According to their survey, 67.4% of the participants had healthy FF, while 25.5% had mild family dysfunction and even 7.1% had severe family dysfunction. Besides, evidence about factors associated with nurses' FF is quite rare. To our best knowledge, a recent study (25) tested factors that might be related to FF, and the result indicated that there was a significant relationship between education background, work unit, and family income with FF among pregnant nurses with second children. However, Tai et al. (28) believed that marital status and shift work predicted the FF in registered nurses. Additionally, some authors thought personality trait was an important factor in predicting FF in nurses (29). However, it is difficult to generalize the conclusion to nurses' FF because of limited reports with a small sample size or one single-center study. In response to those limitations of previous empirical studies, there is a growing need to conduct a study with a large sample size to get a better understanding of the perceived FF in nurses, particularly in China, and to identify relative predictors.

The main purpose of the current study was to gain an insight into the FF among Chinese nurses. The following research questions would be answered: (1) What level of FF was among nurses in China? (2) Which of the observed factors was related to nurses' FF?

## Materials and Methods

### Design, Setting and Participants

A multi-center cross-sectional survey was carried out from January 8^th^, 2020 to January 30^th^, 2020 in 42 hospitals from 26 cities, 16 provinces in China. The hospitals involved were located in the west, southwest, south, central, east, and north of China. In addition, these hospitals, which were representative of different levels of healthcare services, were including primary, secondary, and tertiary institutions. Registered nurses who were working as staff nurses in the selected hospital were included. Nursing students and retired nurses were excluded.

The investigation was completed online. Firstly, researchers imported the informed consent agreement and the questionnaires, Chinese Family Function Scale (CFFS) (30), on an online survey plat, Chinese Questionnaire Star (https://www.wjx.cn/), then freely created a QR code. Researchers released the QR code through the social media application WeChat. Participants could scan the QR to read and submit the informed consent agreement and questionnaires. Each participant was only allowed to submit once in order to avoid double submission.

### Variables and Measurements

Independent variables in the study were some sociodemographic characteristics including gender, age, basic education background, highest education background, hospital level, working years, rotation shift status, working department, marital status, number of children, living with parents, monthly income per family member.

In terms of the primary outcome, CFFS was used since our main purpose was to assess FF in the current study. CFFS was developed by Zhang and Yuan (30). The scale was composed of 18 items. Participants could choose “yes” or “no” to answer each item. In items of 1, 2, 3, 4, 6, 12, 13, 14 and 18, “yes” =1, “no” =0. However, in the other items, “yes” =0, “no” =1. Thus, the total score of the 18 items ranged from 0 to 18. The higher scores, the more severer family dysfunction. The Cronbach's α was 0.873.

### Data Collection

Two researchers who didn't know the study design collected the data. Before the data collection, those two researchers received training courses about how to collect the data, check the data, input and code the data into IBM SPSS 25.0. After the training courses, they took part in an exam related to data collection. Only if they passed through the exam could they be involved in collecting data.

### Data Analysis

All data were imported into IBM SPSS 25.0. In terms of continuous data, tests for normality and homogeneity of variances must be performed first. Mean ± standard deviation (M ± SD) was used to describe if the continuous data were normally distributed, However, median and IQR were used to describe the non-normally distributed continuous variables. Categorical variables were presented as frequency (N) and percentages. In order to test the potential factors associated with FF, a univariate analysis was conducted first. At this stage, we used Spearman's rank correlation analysis to test the correlation between two continuous variables, and the Mann-Whitney U test or Kruskal-Wallis H test was performed to assess the difference of FF scores in different demographic pairs. If the results showed the statistical significance of FF score in demographic pairs, the pairwise comparison method was used to determine whether they were significantly different from each other. After that, categorical regression (optimal scaling regression) was the main method to analyze factors that had been confirmed to be statistically significant in the univariate analysis. In the current study, *P*-value was two-tailed and we inferred statistical significance if α was < 0.05.

## Results

### Demographic Characteristics of the Participants

Nineteen thousand five hundred and twenty-two nurses completed the questionnaires. However, 100 questionnaire answers were removed from the analysis because of the following reasons: (1) Responders' age was logically incorrect; (2) Participants' working years were more than their age. Therefore, the effective response rate was 99.49%.

As shown in Table 1, female nurses dominated the main responders (95.66%). Participants' age ranged from 18 to 62 years old and the median age was 29 years old (IQR:26–33). Most (55.11%) of the included nurses originally graduated from higher vocational school (HVS), while only 4,531 nurses (23.33%) originally graduated from college or university and got a bachelor's degree. However, 54.91% of the included nurses finally got a bachelor's degree after receiving continuous education. Ten thousand four hundred eighty-seven nurses were working at a secondary level of hospital and 8,619 nurses were at a tertiary hospital. In the current study, the working years of nurses ranged from 1 to 41 years and the median of the working years was 6. Nurses who worked rotation shifts were more than 55%. Besides, nurses on rotation shift working were younger, less working age, mainly never married, less educated than those without rotation shift. And more than 70% of the respondents worked at the Inpatient general department. 68.02% of the nurses were married and 28.71% were never married. Only 36.75% of the nurses reported without any child. Nearly 80% of the respondents reported a monthly income per family member of more than 2000 RMB (¥ 2000).

**Table 1:** Demographic characteristic and outcome of univariate analysis (*N* = 19,422).

	**Frequency(%)**	**Mean rank**	**U/H**	***P***
**Gender**
Male	842(4.34)	9803.26	7744916.000	0.624
Female	18,580 (95.66)	9707.34		
**Age**	29.00^a^(21–33) ^b^		0.021^c^	**0.004**
**Basic education level**
Diploma of SVS	4,187 (21.56)	9736.62	0.793	0.673
Diploma of HVS	10,704 (55.11)	99728.77		
Bachelor Degree	4,531 (23.33)	9647.48		
**Highest education level**
Diploma of SVS	495 (2.55)	9821.85	36.514	**<0.001**
Diploma of HVS	8,234 (42.40)	9432.17		
Bachelor Degree	10,664 (54.91)	9920.93		
Master Degree	27 (0.14)	10048.33		
PHD Degree	2 (0.01)	11151.75		
**Hospital level**
Primary or community	316(1.63)	10277.43	69.415	**<0.001**
Secondary	10,487 (54.00)	9997.85		
Tertiary	8,619 (44.38)	9342.34		
**Working years**	6.00^a^(3–11) ^b^		0.016	**<0.031**
**Rotation shift status**
Yes	10,846 (55.84)	9987.72	43511762.5	**<0.001**
No	8,576 (44.16)	9362.17		
**Working department**
Outpatient	2,277 (11.72)	9332.97	31.595	**<0.001**
Emergency	1,165 (6.00)	9789.49		
Inpatient general ward	13,599 (70.02)	9678.57		
ICU	2,381 (12.26)	10223.42		
**Marital status**
Never married	5,577 (28.71)	9353.18	34.462	**<0.001**
Married	13,211 (68.02)	9869.66		
Widowed	47(0.24)	9680.77		
Divorced	587(3.02)	9558.76		
**Number of children**
0	7,138 (36.75)	9198.45	126.504	**<0.001**
1	7,701 (39.65)	9804.82		
2	4,507 (23.21)	10339.44		
3	76 (0.39)	11202.85		
**Living with parents**
Yes	9,852 (50.73)	9646.85	46504885.00	0.990
No	9,570 (49.27)	9778.06		
**Monthly income per family member**
≤550	690(3.55)	10688.62	158.777	**<0.001**
551 1,200	2,185 (11.25)	10283.94		
1,201 2,000	3,005 (15.47)	10078.86		
2,001 3,000	5,025 (25.87)	9891.73		
3,001 6,000	6,142 (31.62)	9475.42		
>6,000	2,375 (12.23)	8665.37		

a *Media*;

b *IQR*;

c *Spearman's Correlation Coefficient (R); SVS, Secondary Vocational School; HVS, Higher Vocational School. Bold values are statistically significant*.

Nurses' perceived FF scores ranged from 0 to 18, and the median was 3 (IQR 2–5). As explained before, the less score on the FF scale, the higher level of FF. In the current study, 1,145 nurses got 0 scores. More than 55% (10,848) of nurses got < 4 scores, and about 96.69% of nurses got FF scores < 11, while 838 (3.31%) nurses got a score greater than or equal to 11. Whereas, only 12 nurses (0.06%) reported 18 scores.

### Univariate Analysis

The outcome of univariate analysis of FF score and pairwise comparison in different demographic pairs was namely presented in Tables 1, 2. Results in the Table 1 showed that age (*R* = 0.021, *P* = 0.004) and working years (*R* = 0.016, *P* = 0.031) might be related to FF scores. The results in Table 1 didn't show the significance of the distribution of FF scores in nurses of different gender (*P* = 0.624), basic education backgrounds (*P* = 0.673), and in nurses who lived with their parents (*P* = 0.990). However, data analysis highlighted that the highest education level, hospital level, rotation shift working, working department, marital status, number of children, monthly income per family member might be factors associated with the difference in FF score distribution in nurses (*P* **<** 0.05) (seen in Table 1).

**Table 2:** The outcome of pairwise comparison in different demographic pairs.

	**Test statistics**	**Adjusted *P***
**Highest education level**
Diploma of HVS-Diploma of SVS	389.673	1.000
Diploma of HVS-Bachelor Degree	−488.759	**<0.001**
Diploma of HVS-Master Degree	−616.161	1.000
Diploma of HVS-PHD Degree	−1719.577	1.000
Diploma of SVS-Bachelor Degree	−99.087	1.000
Diploma of SVS-Master Degree	−226.488	1.000
Diploma of SVS-PHD Degree	−1329.905	1.000
Undergraduate-Master Degree	−127.401	1.000
Undergraduate-PHD Degree	−1230.818	1.000
Postgraduate-PHD Degree	−1103.417	1.000
**Hospital level**
Tertiary-secondary	655.509	**<0.001**
Tertiary-primary or community	935.091	**0.010**
Secondary-primary or community	279.582	1.000
**Working department**
Outpatient-inpatient general ward	−345.602	**0.036**
Outpatient-emergency	−456.528	0.134
Outpatient-ICU	−890.458	**<0.001**
Inpatient general ward-emergency	110.926	1.000
Inpatient general ward-ICU	−544.856	**<0.001**
Emergency-ICU	−433.93	0.172
**Marital status**
Never married-divorced	−205.584	1.000
Never married-widowed	−327.589	1.000
Never married-married	−516.485	**<0.001**
Divorced-widowed	122.005	1.000
Divorced-married	310.901	1.000
Widowed-married	188.896	1.000
**Number of children**
0–1	−606.372	**<0.001**
0–2	−1140.988	**<0.001**
0–3	−2004.398	**0.01**
1–2	−534.616	**<0.001**
1–3	−1398.025	0.173
2–3	−863.41	1.000
**Monthly income per family member**
>6,000–3,001 6,000	810.047	**<0.001**
>6,000–2,001 3,000	1226.361	**<0.001**
>6,000–1,201 2,000	1413.488	**<0.001**
>6,000–551 1,200	1618.571	**<0.001**
>6,000–≤550	2023.252	**<0.001**
3,001 6,000–2,001 3,000	416.314	**0.001**
3,001 6,000–1,201 2,000	603.442	**<0.001**
3,001 6,000–551 1,200	808.524	**<0.001**
3,001 6,000–≤550	1213.205	**<0.001**
2,001 3,000–1,201 2,000	187.128	1.000
2,001 3,000–551 1,200	392.21	0.087
2,001 3,000–≤550	796.891	0.006
1,201 2,000–551 1,200	205.082	1.000
1,201 2,000–≤550	609.763	0.138
551 1,200–≤550	404.681	1.000

*Bold values are statistically significant*.

Table 2 revealed the outcome of pairwise comparison if there was significance in the distribution of FF score among 3 groups of the demographic variables. There was a difference in the FF score in nurses with diplomas of HVS and with bachelor's degrees, and the difference was statistically significant (Adjusted *P* **<** 0.001). However, we did not detect any significant differences in the FF score in nurses when conducting a pairwise comparison between other two different highest education levels (Table 2). In addition, the level of FF score was significantly different between nurses working in tertiary and secondary levels of the hospital (Adjusted *P* **<** 0.001), tertiary and primary/community hospitals (Adjusted *P* = 0.010). In terms of rotation shift working, we found that the mean rank of FF score in nurses working rotation shift was 9987.72, while it was 9362.17 in nurses who were not working rotation shift, and the difference was statistically significant (*P* **<** 0.001). In addition, the FF score in nurses working rotation shifts was higher than those not working rotation shifts. Results in Table 2 indicated that there was a significance in the level of FF score in nurses who worked in outpatient vs. inpatient general ward (*P* = 0.036), outpatient vs. ICU (*P* **<** 0.001), and inpatient general ward vs. ICU (*P* **<** 0.001). Compared with nurses with children, nurses without children had less FF scores, and the difference of that was also detected in a pairwise comparison (Table 2). In the current study, we also found that FF scores varied in nurses with different monthly incomes per family member, the higher monthly income per family member, the less FF scores, especially when nurses' families got monthly income per family member of more than ¥ 3000, and the difference was significant (Table 2).

### Multivariate Analysis

The result of the multivariate analysis, presented in Table 3, showed that the highest education level, hospital level, rotation shift working, working department, number of children nurses had, and monthly income per family member were included in a regression model, and the model was significant (*P* < 0.001). However, the regression model did not include age (*P* = 0.050), working years (*P* = 0.898), and marital status (*P* = 0.051) although they were significantly associated with FF score when conducting univariate analysis. The outcome of importance was summarized in Table 4. Notably, the result pointed to the importance of the highest education level, hospital level, rotation shift working, working department, the number of children, and monthly income per family member in predicting nurses' perceived FF score in such a regression model. In particular, the number of children was the dominant factor in predicting the FF score (49.1%). Monthly income per family member was another important factor to predict the FF score (20.7%). The importance of other independent variables in predicting FF score, in turn, was rotation shift working status (12.4%), the highest education level (8%), the hospital level (7.6%), and working department (2.4%).

**Table 3:** The outcome of coefficients table.

	**SC**			**F**	***P***
	**Beta**	**SE of Beta**	**df**		
Age	−0.039	0.020	1	3.829	0.050
Highest education level	0.052	0.007	3	52.291	**<0.001**
Hospital level	−0.046	0.007	2	39.331	**<0.001**
Working years	−0.003	0.020	1	0.017	0.898
Rotation shift status	−0.071	0.008	1	72.691	**<0.001**
Working department	0.026	0.007	3	14.368	**<0.001**
Marital status	−0.012	0.008	3	2.590	0.051
Number of children	0.155	0.012	3	170.248	**<0.001**
Monthly income per family member	−0.081	0.008	5	108.718	**<0.001**

*Dependent variable: FF score; SC, Standardized Coefficients; SE, Standardized error; df, Degree of Freedom. Bold values are statistically significant*.

**Table 4:** The outcome of Correlation and Tolerance.

	**Correlation**			**Importance**	**Tolerance**	
	**Zero-order**	**Part**	**PC**		**BT**	**AT**
Age	−0.013	−0.012	−0.012	0.017	0.096	0.097
Highest education level	0.047	0.050	0.050	**0.080**	0.912	0.915
Hospital level	−0.049	−0.046	−0.045	**0.076**	0.946	0.942
Working years	−0.022	−0.001	−0.001	0.002	0.101	0.101
Rotation shift status	−0.052	−0.065	−0.064	**0.124**	0.800	0.750
Working department	0.027	0.027	0.026	**0.024**	0.993	0.900
Marital status	0.053	−0.009	−0.009	−0.021	0.536	0.629
Number of children	0.095	0.104	0.103	**0.491**	0.438	0.617
Monthly income per family member	−0.077	−0.076	−0.075	**0.207**	0.864	0.863

*Dependent variable: FF score; PC, Partial correlation; BT, Before transformation; AT, After transformation. Bold values are statistically significant*.

## Discussion

The current study provided empirical evidence for FF in registered nurses. Our study examined the FF of nurses and identified some relative factors which might predict nurses' perceived FF scores. We recruited 19,522 registered nurses in this descriptive cross-sectional study. Nineteen thousand four hundred twenty-two nurses finally completed the online questionnaires. The results showed most of the nurses who participated in the study demonstrated appropriate FF. The result was in accordance with the study conducted by Guo (31).

The present study explored factors that might be predictors of nurses' perceived FF. The final results showed that the highest education level, hospital level, rotation shift status, working department, number of children, and monthly income per family member were associated with FF scores.

Interestingly, our study highlights the importance of the number of children. The result indicated that the number of children was the most important factor with an importance ratio of 49.1% in predicting nurses' FF. Compared to nurses without any children, the FF score was higher in nurses having children, which indicated less level of FF. In addition, our study showed that the more children nurse had, the less level of FF. On one hand, Chinese nurses experience a higher level of workload than nurses in developed countries. According to WHO's World Health Statistics 2020 (32), the density of nursing and midwifery personnel (per 10,000) was 26.6 in China, while that was 81.7 in the United Kingdom, 99.4 in Canada, 145.5 in the United States of America. On the other hand, nurses, especially female nurses, are still the main force of taking responsibility for caring for and educating children in their families in China (33). The reasons above make it sense that a higher level of work-family conflict was reported in subjects who had dependent care responsibilities for child/children because of inflexible commitments at home or less level of control over work when arranging childcare and caring for sick child/children (34). Compared to nurses having one child, nurses having more than one child would face a higher level of challenges in balancing work and family demands. As a result, they might involve in a lower level of satisfaction with work-family responsibilities and less level of FF (35). The Chinese government issued a universal two-child policy in 2015 (36), while issued the third-child policy in 2021 to encourage births (37). However, the supporting measures, especially for supporting daycare of children before attending kindergarten for income households still need to be improved. In other countries like Japan, the government releases childcare services covering 0–5 years old children (38) to reduce work-family conflicts for residents. Thus, the current study suggests government, Policymakers, and hospitals should take measures to promote children-care services for nurses.

One of our key findings was a significant relationship between monthly income and nurses' perceived FF. There are some ongoing debates on the relationship between monthly income and FF. Some scholars believed that monthly income was one of the influence factors of FF (1, 39). One possible reason might be that work interference with the family occurs less in families with greater monthly income (35). However, some authors argued that monthly income was not associated with work-family conflict (40). Our study offered an insight into the relationship between monthly income and FF in nurses. In the current study, we analyzed whether monthly family income, especially monthly income per family member, would be a predictor of nurses' perceived FF. Similarly, our results showed that monthly income per family member, with an importance ratio of 20.7%, was one of the most important factors predicting nurses' FF. The result is consistent with Dai and Wang's study (1, 39). Additionally, the results also indicated that nurses with a higher level of monthly income per family member showed a higher level of perceived FF. According to national statistical data, the annual mean wage of Chinese health care workers was 115,449 Yuan (< $20,000) in 2020 (41), which was much less than that of American registered nurses whose annual wage ranged from $59,450 to $120,250 (42). However, the nurse density per 10,000 population in China was 31.4 (43), while that in America was 83.4 (44). In other words, Chinese nurses' income was much more disproportionate to the intensity of their workload than that of developed countries. Therefore, we suggest that measures should be taken by policymakers and employers to increase nurses' income in order to improve their FF.

In the present study, 55.84% of participants were working rotation shifts. According to the results of our study, shift working was found as an important factor associated with nurses' perceived FF score. Compared to dayshift nurses, rotation shift or evening shift nurses got higher FF scores in the study, which implied less level of FF. Our findings corroborated with the result of Tai et al. (28). They did a survey in 1,438 registered nurses and found that the FF was poor in nurses who were on rotation shift than those on dayshift (OR = 1.38, 95%CI: 1.01–1.88). Several studies have been done to explore whether there is a relationship between shift working and work-family conflict in nurses (35, 45–47). There is an ongoing debate in shift working and the work-family conflict. Some authors (47) highlighted that, for night shift nurses, the effect of the night shift on the conflict between work and childcare was not significant. Some other authors argued that irregular work schedule was one of the main contributors to work-family conflict. To our knowledge, nurses provide health services for clients, and such a job is perceived as a high-demand job. In other words, nursing is a job with overtime, intensity, irregular work schedule, inflexible work (46). In particular, nurses have to work shifts in order to ensure continuous health care for patients (48). Additionally, nurses usually experience irregular sleeping times. Besides, nurses rarely take a normal break on holidays. Thus, nurses spend less time being together with family or communicating with their relatives, which may increase the risk of disruption to social and family interactive life (28), which may lead to a less level of FF. On one hand, as described above, the nurse density per 10,000 population in China was 31.4 (43), which demonstrated that there was a large shortage in the nursing workforce in China. On the other hand, there was a high rate of nurse turnover (49). As a result, nurses in the clinical settings had to work with more night shift rotations. Therefore, strategies should be implemented to solve the deficit of nursing staff, and to educate and train enough nurses to meet the demands of healthcare system (50), as well as to improve nurses' FF.

The association between education level and FF is also demonstrated in this study. Interestingly, we did not find any relationship between basic education level and FF. Whereas, there was a significant relationship between the highest education level. Comparatively, nurses with a Master's degree or PH.D. got higher FF scores. In other words, nurses with higher education backgrounds might experience poor FF. In China, nurses' basic education was classified into three levels: secondary vocational school, higher vocational school, and bachelor's degree. Nurses could gain higher education levels through continued education. With greater nursing education levels, nurses might face more frequent family interference with work (35). Yu revealed that, in clinical settings, nurses with a higher education level worked in many different roles. They had to complete the clinical work as a caregiver, communicator, educator et al. they may also be under high pressure with scientific research. Hence, they could not make a good balance between work and family, on one hand, resulting in poor feelings in their family roles (25). On the other hand, that made it difficult to satisfy family-related responsibilities (35). We supposed that such a problem was due to a big gap in career development for clinical nurses, especially for clinical research nurses in China. In China, nurses with master's degrees or higher education always are assigned research masks. However, the development of clinical research nurses was in the initial stage so there was a lack of relative rules or regulations to define or clarify the job of clinical research nurses (51). Therefore, our result suggested that Policymakers and nursing managers should design clear plans and specific strategies to build a well-established system in order to reach nurses' best potential and improve their FF.

Based on the results of the study, the hospital level might also play an important role in influencing nurses' FF. In China, China's Ministry of Health classified the hospital level into Primary (level 1), Secondary (level 2), and Tertiary (level 3) hospitals based on the beds number of a hospital (52). Yu's study also addressed the relationship between the hospital level and FF. They found that nurses working in the Tertiary hospital faced a higher level of family-work conflict because of higher job-related pressure, leading to inappropriate FF (25). However, our study indicated that the FF of nurses working in higher-level hospitals was better. We supposed that, in China, the average income of nurses working in higher-level hospitals was much better than those in lower-level hospitals. As the above data showed that there was a significant association between monthly income and FF. Hence, the FF of nurses working in the Tertiary hospital was better than others in Secondary and Primary hospitals in our study.

The relationship between the working department and FF was also tested in this study. The result verified the significant association between these two variables. Specifically, it is notable that nurses working in the ICU got the highest FF score, followed by those working in the Emergency, inpatient general ward, and outpatient departments in turn. Specifically, previous research documented that ICU was a place where the nursing staff was stressful and challenging because of staff shortage, the complex nature of patients' conditions, frequent rescue, overload working, high expectations from administrators, sophisticated technology systems, ethical dilemmas in dealing with death, as well as inadequate income (53). Nurses in the ICU environment may feel a higher level of stress and work-family conflict than nurses working in other units. Therefore, they may experience a higher level of family dysfunction. Comparatively, outpatient nurses gained a higher level of FF, which might be explained by that they don't need to work rotation shifts. Additionally, patients in the outpatient department usually are with chronic illnesses or in less critical conditions. Thus, outpatient nurses are under less stress so they could handle work-family conflict much better than nurses working in other units.

In previous studies, scholars found that FF decreased in nurses who worked for longer years (31). They supposed that nurses with longer working years lived with some degree of social status and a stable living environment. They are faced with much less sense of crisis so they might take negative coping strategies when dealing with family-related conflicts. In the current study, the relationship between working years and FF was not significant according to the result of the regression, even though that was significant in the univariate analysis.

## Limitations and Recommendations for Future Studies

There are several limitations that need to be acknowledged in the study. First and foremost, due to the nature of the cross-sectional design, we could not gain more and further data, especially about nurses' feelings or experiences on FF because of lacking qualitative evidence in this study. The mixed methodology could be used in future studies to take a deeper insight into nurses' FF. Second, the study is conducted only in China, there are reasons to believe that the results may be different in other countries due to cultural diversity and differences. Future studies could collect data from nurses in different countries to explore cultural-related factors in predicting FF.

## Conclusions

The current cross-sectional study explored Chinese nurses' perceived FF level and the associated predictors. The result showed that most of the nurses who participated in the study demonstrated appropriate FF. Moreover, the study also demonstrated that the highest education level, hospital level, rotation shift status, working department, number of children, and monthly income per family member were significantly associated with nurses' perceived FF. The importance of those predictors was, in turn, the number of children, monthly income per family member, rotation shift status, the highest education level, the hospital level, and the working department.

According to the results of the study, FF was associated with multiple factors, which hints that managers, leaders, and government could make strategies to improve nurses' FF in order to lead nurses to make the balance between family and work. In that way, nurses could make it realized to achieve success of both their nursing careers and in their personal families. Therefore, we suggested that policymakers, nursing managers, and employers should make strategies such as promoting children-care services, increasing nurses' income, educating and training enough nurses, and building a well-established system of career development to help clinical nurses improve their FF so that to improve the quality of care.

## Data Availability Statement

The original contributions presented in the study are included in the article/supplementary material, further inquiries can be directed to the corresponding authors.

## Ethics Statement

Under the guidance of principles of the World Medical Association Declaration of Helsinki (54), it was taken into the first consideration to respect participants' rights and to protect their health and rights. The investigation was an anonymous survey. Informed consent was delivered verbally when participants were included and accessed online. Since the current study was a cross-sectional study in which the only risk was about participants' privacy, when conducting the survey, the participants would be informed about the purpose of the study, the way and the time of conducting the survey, their rights to refuse the survey, and how their information was stored, protected, and used in an academic way. The study was approved by the Ethical Committee of the corresponding institution, Guizhou Provincial People's Hospital (reference 2018072).

## Author Contributions

WZ, QF, SS, and YY conceived and designed the study. CW and DL collected input and checked the data. BT, ZZ, and PL analyzed the data. WZ drafted the manuscript. All authors read and approved the final manuscript.

## Funding

This study was funded by the Ministry of Science and Technology of the People's Republic China (2020YFC2008801 and 2020YFC2008800), National Natural Science Foundation of China, Guizhou (81860245), and Science and Technology Platform and Talent Team Planning Project ([2019]5664), which offered support in the process of study design and financial support for publication.

## Conflict of Interest

The authors declare that the research was conducted in the absence of any commercial or financial relationships that could be construed as a potential conflict of interest.

## Publisher's Note

All claims expressed in this article are solely those of the authors and do not necessarily represent those of their affiliated organizations, or those of the publisher, the editors and the reviewers. Any product that may be evaluated in this article, or claim that may be made by its manufacturer, is not guaranteed or endorsed by the publisher.
